# Draft Genome Sequence of the Environmental Isolate *Chryseobacterium* sp. Hurlbut01

**DOI:** 10.1128/genomeA.01071-15

**Published:** 2015-09-17

**Authors:** M. B. Couger, Allison Hurlbut, Chelsea L. Murphy, Connie Budd, Donald P. French, Wouter D. Hoff, Mostafa S. Elshahed, Noha H. Youssef

**Affiliations:** a Department of Microbiology and Molecular Genetics, Oklahoma State University, Stillwater, Oklahoma, USA; b Department of Integrative Biology, Oklahoma State University, Stillwater, Oklahoma, USA; c Department of Chemistry, Oklahoma State University, Stillwater, Oklahoma, USA

## Abstract

We report here the draft genome sequence of the environmental isolate *Chryseobacterium* sp. Hurlbut01, isolated from a light switch surface in Stillwater, OK, as part of the Student-Initiated Microbial Discovery (SIMD) project. The genome has a size of 3,899,838 bp and a contig *N*_50_ of 321 kb.

## GENOME ANNOUNCEMENT

Participation in research has a strong influence on undergraduate persistence in science, technology, engineering, and mathematics (STEM) (1–3). However, providing research opportunities, particularly for first- and second-year students, is challenging (4, 5). Vast, unexplored, microbial diversity and the availability of modern DNA sequencing and genome analysis techniques provide exciting opportunities for involving undergraduates in current research. Here, we report the first genome sequence for a bacterium isolated by an undergraduate (A. H.) in an introductory microbiology course modified to be the initial course in our microbial discovery and genome analysis sequence. This sequence is part of a project at Oklahoma State University funded by the Howard Hughes Medical Institute aimed at improving student persistence through authentic undergraduate research.

The genome of *Chryseobacterium* sp. strain Hurlbut01 was sequenced using the Illumina MiSeq platform at the University of Georgia Genomics Facility using 2 × 300 paired-end chemistry and an average library insert size of 700 bp. Quality-filtered sequence data were assembled with the short-read de Bruijn graph (6) assembly program Velvet (7). The assembly settings were a *k*-mer value of 101 bp and a minimum contig coverage value of 7×. The genome assembly process produced a contig *N*_50_ of 320,779 bp, with a total genome size of 3,899,838 bp (3.90 Mb). The G+C content was 34.05%. Gene models were created using the prokaryotic gene calling software package Prodigal (8). A total of 3,643 gene models were predicted. The average gene size was 954 bp. Translated protein sequences were functionally annotated using a combination of NCBI BLAST C++ homology search (9) and HMMER 3.0 hmmscan (10, 11) against the Pfam 26.0 database (12).

A 16S rRNA gene sequence comparison was used to identify strains with sequenced genomes that are most closely related to *Chryseobacterium* sp. strain Hurlbut01. Strain Hurlbut01 was 98.0% similar to *Chryseobacterium soli* DSM 19298, 96.0% similar to *Chryseobacterium* sp. StRB126, *Epilithonimonas lactis* strain LMG 24401, *Epilithonimonas* sp. FH1, and *Flavobacteriaceae* bacterium 3519-10, and 95.0% to *Riemerella anatipestifer* CH3. Among 3,301 predicted proteins in strain Hurlbut01, 1,535 (46.5%) were most similar to *C. soli*, 989 (30.0%) were most similar to *Chryseobacterium* sp. StRB126, 326 (9.9%) were most similar to *E. lactis* LMG 24401, 238 (7.2%) were most similar to *Epilithonimonas* sp. FH1, 142 (4.3%) were most similar to *Flavobacteriaceae* bacterium 3519-10, and 70 (2.1%) were most similar to *R. anatipestifer* CH3. A number of *Chryseobacterium* sp. strain Hurlbut01 genes (428/3,738 [1.28%]) have no significant similarity to these closest 16S rRNA relative genomes (i.e., had e-value >10^-5^ to closest 1st-hit relative in genome-to-genome BLAST-p comparisons). Further, a comparison of the *Chryseobacterium* sp. strain Hurlbut01 genome versus all available *Chryseobacterium* genomes (*n* = 17 in NCBI Genomes as of June 2015) identified 233 genes that are unique to this strain. These genes were involved in B12 biosynthesis, capsular protein production, xenobiotic response elements, and numerous hypothetical proteins. These initial results highlight the high level of intralineage diversity within members of this genus despite the relatively high level of 16S rRNA gene sequence similarity.

### Nucleotide sequence accession number.

The GenBank accession number for the genome is LGIP00000000.
